# Curcumin Encapsulated Lecithin Nanoemulsions: An Oral Platform for Ultrasound Mediated Spatiotemporal Delivery of Curcumin to the Tumor

**DOI:** 10.1038/s41598-020-65468-1

**Published:** 2020-05-22

**Authors:** Chandrashekhar Prasad, Eshant Bhatia, Rinti Banerjee

**Affiliations:** Department of Biosciences and Bioengineering, Indian Institute of Technology Bombay, Mumbai, India

**Keywords:** Drug delivery, Drug delivery

## Abstract

Systemic toxicity caused by conventional chemotherapy is often regarded as one of the major challenges in the treatment of cancer. Over years, the trigger-based modality has gained much attention as it holds the spatiotemporal control over release and internalization of the drug. In this article, we are reporting an increase in the anti-tumor efficacy of curcumin due to ultrasound pulses. MDA MB 231 breast cancer and B16F10 melanoma cells were incubated with lecithin-based curcumin encapsulated nanoemulsions and exposed to ultrasound in the presence and absence of microbubble. Ultrasound induced sonoporation enhanced the cytotoxicity of curcumin in MDA MB 231 and B16F10 cancer cells in the presence of microbubble by 100- and 64-fold, respectively. To study the spatiotemporal delivery of curcumin, we developed B16F10 melanoma subcutaneous tumor on both the flanks of C57BL/6 mice but only the right tumor was exposed to ultrasound. Insonation of the right tumor spatially enhanced the cytotoxicity and enabled the substantial regression of the right tumor compared to the unexposed left tumor which grew continuously in size. This study showed that the ultrasound has the potential to target and increase the drug’s throughput to the tumor and enable effective treatment.

## Introduction

Cancers are heterogenous diseases characterized as uncontrolled, random cell division and invasiveness of abnormal or genetically manipulated cells. 5 to 10% of cancers are genetically inherited but majority of cancers are the results of environmental factors, such as prolonged exposure to radiation and pollutants, unhealthy lifestyle including tobacco smoking, and stress^1,2^. According to a report of the World Health Organization, 9.6 million people died due to cancer in 2018, and the disease became the second most leading cause of death in that year. This statistic reflects a scope of improvement in the current modalities of treatment. The current modalities of treatment include cytotoxic chemotherapy, targeted therapy, endocrine therapy, immunotherapy and radiation therapy. These treatment modalities have their own limitation with regard to effective treatment of cancer and rate of survival of patients. Although, achievements have been made in the past decades in the treatment modality but non-specificity of the conventional chemotherapy towards a specific tumor and the emerging drug resistivity of the tumor are the major challenges. Moreover, the solid tumor with distant blood vessels, the complex composition of extracellular matrices, cell-cell adhesion, high interstitial fluid pressure and lack of convection often makes elusive for the drug to penetrate deeper inside the tumor and thus, only subtherapeutic doses can effectively reach the core of tumor^3^. The heterogeneity of the tumor further leads to variable effects of drugs in different patients. In recent decades, nanomedicine has shown great prospects in augmenting drug delivery thus, achieving high therapeutic concentrations of the drug inside the tumor even after the administration of a suboptimal dose^4^. One such method is the use of external trigger which is intended to focus over the tumor and selectively increase the throughput of a drug to the tumor^5,6^. Various external triggers such as temperature, magnetic field, ultrasound and light have been employed by researchers to enhance the effects of nanocarriers for cancer therapy^7,8^.

Curcumin is a plant (*Curcumin longa*) derived anti-inflammatory, antibacterial, antioxidant and anticancer agent with a very minimal side effect. In phase II clinical trial(s), Dhillon et al. found that 8 g/day dose of oral curcumin was safe and well-tolerated in patients with advanced pancreatic cancer for up to 18 months but very low detectable systemic level of curcumin (22–41 ng/ml of plasma) was achieved suggesting poor absorption and bioavailability^9^. In colorectal carcinoma patients, when 3.6 gm of oral curcumin was administered every day, only few nanomolar concentrations of curcumin and its glucuronides were detected in peripheral blood and portal circulation^10^. Though, there are studies about the effectiveness of curcumin, very high doses are required because of its low solubility and bioavailability^11^. Curcumin has been encapsulated inside polymer or lipid based colloidal particles, such as liposomes^12^, emulsions^13^, nanoemulsions^14^, biopolymeric microgels^15^, solid lipid nanoparticles^16^, micelles^17^ and polymeric nanoparticles^18^ which are dispersed in the aqueous medium. Encapsulation of curcumin inside these nanoparticles increased the solubility and bioavailability. However, to the best of our knowledge, the IC_50_ concentration of curcumin had been reported mostly in the range of micromolar (>1 µM). Lin et al. found that the IC_50_ concentration of the cationic liposome-PEG-PEI complex encapsulated curcumin was 1–1.7 µM for a series of cancer cells which was substantially lower than the IC_50_ concentration (7.9–30 µM) of non-encapsulated curcumin^12^. As only a few nanomolar of curcumin was found in the blood even after the administration of 8 g/day, it is a prerequisite to reduce the IC_50_ of the drug towards cancer cells to induce effective treatment of tumor. An ultrasound trigger can be employed to increase the cytotoxicity of curcumin as well as increase the spatial delivery of cargo to the tumor site and minimize the systemic toxicity.

Ultrasound has been used as a non-invasive treatment method in cancer therapy. Generally, three main effects of ultrasound on tumor are seen namely- mechanical, temperature and cavitation. High intensity ultrasound relies majorly on thermal effects, which are not easy to control^19^. The therapeutic benefits of low intensity ultrasound, on the other hand, is based on cavitation effect. When ultrasound waves travel through a liquid medium, it results in expansion, and contraction of microbubbles used as ultrasound contrast agents and lead to cavitation or bursting of the microbubbles that temporarily causes mechanical damage to the plasma membrane of cells in the vicinity^20^.

Microbubbles are gas containing contrast agents, where in low solubility perfluoro gas is surrounded by a phospholipid shell. Forced expansion and contraction of microbubbles leads to their destruction thus affecting the permeability of cell membrane^21,22^. With the rapid development of molecular imaging technology, nanoscale ultrasound contrast agents, such as nanoliposome contrast agent and nano-fluorocarbon emulsion, have emerged in recent years^23,24^.

In our previous work, we have developed microbubbles-nanocapsule conjugates that served as better contrast agents for ultrasound mediated imaging as compared to Sonovue, a commercial ultrasound contrast agent, for enhanced delivery of hydrophilic and hydrophobic drugs to tumor, where we observed that the presence of microbubbles enhanced the ultrasound mediated transient pore formation in cancerous cells and increased the cellular internalization^25^.

Based on similar rational of using ultrasound responsive microbubbles for cancer therapy, we have combined ultrasound treatment with oral drug delivery of curcumin. In the current study, we explore the spatiotemporal delivery and anti-tumor activity of the oral curcumin formulation followed by intravenous administration of sulfur hexafluoride (SF_6_)-containing-lipid-shelled-microbubbles (MB) and ultrasound exposure of the tumor. The oral formulation, curcumin encapsulated lecithin nanoemulsions (Cur_NE) was developed and then *in vitro* cytotoxicity was studied in cancer cells: MDA MB 231 and B16F10. *In vivo* anti-tumor efficacy was studied in the B16F10 subcutaneous tumor model developed on both right and left flank of C57BL/6 mice, but the only right tumor was exposed to ultrasound. The reduction in volume of the tumor exposed to ultrasound was compared with size reduction in the tumor of the same mice (left tumor) not exposed to ultrasound.

## Results

### Physiochemical characterization of Cur_NE and MB

**Figure 1:**
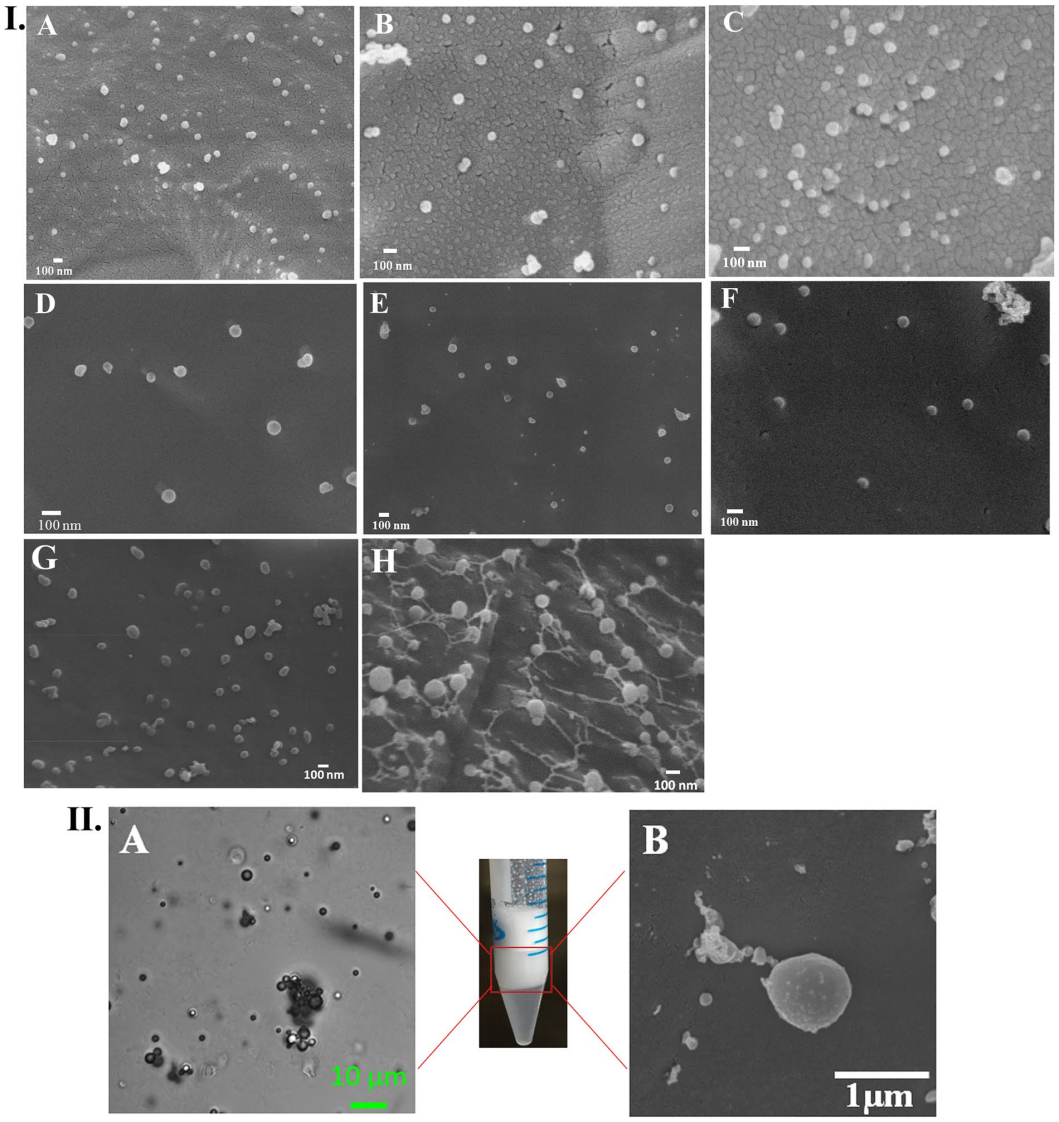
I. Cryo FEG SEM image of Cur_NE with different ratio of lecithin and curcumin respectively (**A**) 27:1 (**B**) 9:1 (**C**) 6:1 (**D**) 4:1 (**E**) 3:1 (**F**) 2:1 (**G**) 1.5:1 (**H**) 1:1. II. Microscopic image of MB (**A**) light microscope (**B**) Cryo FEG SEM.

The hydrodynamic diameter and zeta potential of the Cur_NE are summarized in Table 1. Cur_NE were found to have a hydrodynamic diameter of 60–120 nm. The Cur_NE were also characterized for its size and morphology through a scanning electron microscope and it had shown to be spherical in size with a diameter varying from 50–120 nm, which is in agreement with the hydrodynamic diameter obtained in dynamic light scattering measurement (Fig. 1I). Moreover, the zeta potential of the Cur_NE with different ratio of lecithin and curcumin did not change substantially and was decreasing from −20 to −45 mV (Table 1) which says that particles are stable in the colloidal state in an aqueous medium as the negative zeta potential would substantiate the repulsive forces present between particles and prevent their flocculation^26^. The encapsulation efficiency of the curcumin in Cur_NE increased gradually with the increase in curcumin concentration, as summarized in Table 2. The increase in concentration of curcumin induced a very nominal increase in size of Cur_NE perhaps due to preparation under high pressure homogenization. But the loading capacity of the formulation saturated eventually with the gradual increase in the concentration of the curcumin presumably due to lack of the intra-nanoemulsion space or carrying capacity; and after a specific concentration, encapsulation did not increase further (see Supplementary Fig. S3), rather drug remained free in supernatant and that is why encapsulation efficiency reduced for the ratios 1.5:1 and 1:1 (Table 2). EE (%) of the NERh6g for the Rh6g was found to be 67.5 ± 3.2%. The hydrodynamic diameter of the MBs was found to be 1200.6 ± 201.8 nm and zeta potential −15.9 ± 4.8 mV, which says that MBs are stable in aqueous solution (Table 1). Electron and light microscopy study revealed the spherical shape of MB in an aqueous medium with 900–1000 nm diameter (Fig. 1II) which is in agreement with the size obtained in DLS.

**Table 1:** Size and zeta potential of Cur_NE comprised different ratio of lecithin and drug.

Lecithin: Curcumin (wt: wt)	Size (nm)	Zeta potential (mV)
27:1	60.4 ± 10.3	−36.3 ± 5.2
9:1	92.2 ± 6.4	−26.6 ± 7
6:1	89.5 ± 3.5	−24 ± 5.8
4:1	85.8 ± 4.3	−23.4 ± 6.7
3:1	106.4 ± 3.7	−42.2 ± 10
2:1	110.4 ± 5.3	−34 ± 2.9
1.5:1	115.5 ± 3.2	−40.3 ± 4.3
1:1	118.3 ± 4.2	−38.5 ± 3.8

**Table 2:** Encapsulation efficiency of the Cur_NE with varying ratio of lecithin and curcumin.

Lecithin: Curcumin (wt: wt)	Encapsulation efficiency (%)
27:1	77.0 ± 1.4
9:1	87.7 ± 1.7
6:1	94.9 ± 1.24
4:1	97.8 ± 0.13
3:1	98.0 ± 0.84
2:1	98.8 ± 0.17
1.5:1	88.7 ± 1.7
1:1	82 ± 2.74

### Chemical stability and solubility of curcumin

**Figure 2:**
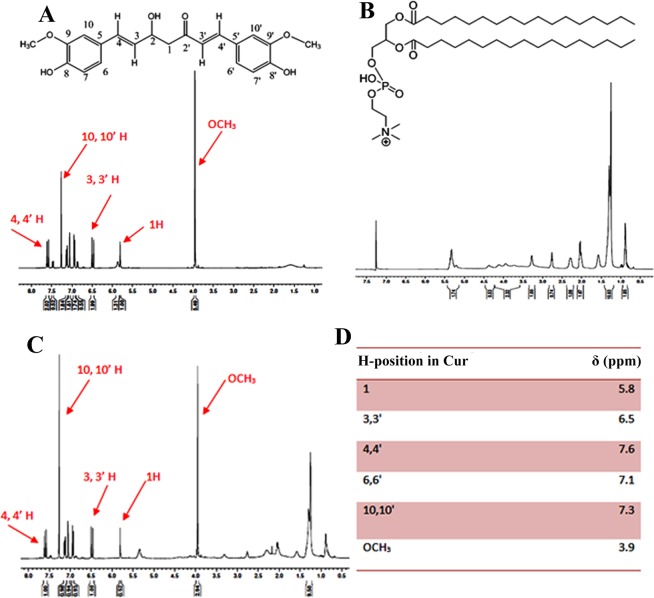
Proton NMR spectrum of (**A**) free curcumin (**B**) lecithin (**C**) Cur_NE (**D**) chemical shift (δ) of protons of curcumin.

The encapsulated curcumin was characterized for its chemical stability with ^1^H NMR spectroscopy and the spectrum of the encapsulated drug was compared with free drug. The chemical shift of the curcumin, both encapsulated and free form is shown in Fig. 2. The existence of the same ^1^H spectra for the free drug as well as drug released after lysis of the Cur_NE indicates that curcumin was chemically stable inside Cur_NE and the process of encapsulation was not altering its natural structure. It is evident from the result that the Cur_NE was soluble in PBS while the same concentration of the free drug was lying over the interface between air and liquid (Supplementary Fig. S3II) whereas the same concentration of encapsulated drug in the form of Cur_NE got easily re-dispersed in PBS without requiring any mechanical agitation or sonication (Supplementary Fig. S3II).

### Gastric resistance and release kinetics in SIF

**Figure 3:**
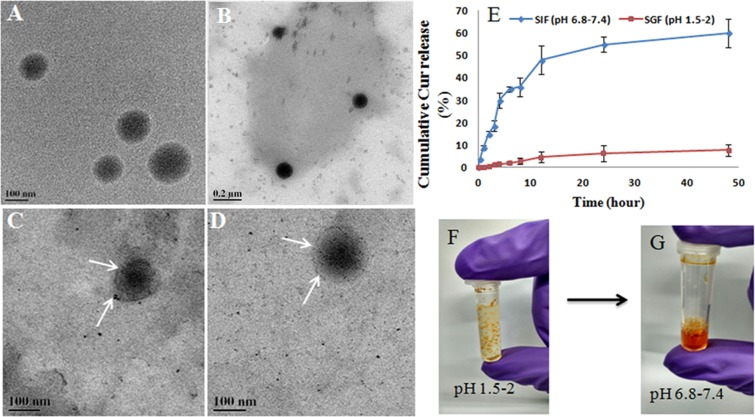
(**A**) Cur_NE without any treatment with SGF or SIF, (**B**) Cur_NE incubated in SGF (pH 1.5–2) for 48 hrs and then pH was adjusted to 6.8–7.4. After that, it was isolated and characterized by FEG TEM, (**C**,**D**) Cur_NE suspended in SIF (6.8–7.4) for 48 hrs and then nanoemulsions were isolated and characterized by FEG TEM. (**E**) Release kinetics of curcumin from Cur_NE in SGF and SIF. (**F**) Agglomeration of Cur_NE occurred in SGF and (**G**) addition of NaOH resuspended the Cur_NE in solution.

The Cur_NE released 0.8 ± 0.08% of encapsulated curcumin within 2 hrs of incubation in SGF and retained >98% of curcumin (Fig. 3E). It showed that the Cur_NEs were resistant to the gastric fluid and therefore encapsulated curcumin would be available for release and absorption in the small intestine. After 48 hrs incubation in SGF, Cur_NEs were still intact and supported the data that showed only 0.8 ± 0.08% of drug released from nanoemulsions within 2 hrs as Cur_NEs were still intact and morphologically stable (Fig. 3B). In SIF the Cur_NE released 30 ± 3.2% of the encapsulated content in 4 hrs (Fig. 3E) and it was evident from the TEM image of the SIF digested Cur_NEs that have diffused core and periphery (Fig. 3C,D). Diffused core and periphery obtained due to the presence of pancreatin and bile salt in SIF that digested the lecithin.

### Cellular internalization of Cur_NE without ultrasound

Cellular internalization study showed that free curcumin was internalized meagrely by cells with an internalized amount of just 0.15 ± 0.05 µg whereas curcumin in the form of Cur_NE was getting internalized in a substantially higher amount of 7.6 ± 1.32 µg within 1.5 hrs of incubation. As we increased the concentration of curcumin in lecithin: drug ratio, internalization was increasing with the increase in the concentration of curcumin to the extent where ratio reached 2:1 but after that, it decreased (see Supplementary Fig. S6). Out of all the ratios, Cur_NE synthesized from 2:1 ratio of lecithin and curcumin showed the highest internalization and it was 50 times higher to free curcumin. Therefore, this ratio was considered for *in vitro* cytotoxicity and *in vivo* anti-tumor efficacy study.

### MB induced sonoporation in MDA MB 231 and B16F10 cells

**Figure 4:**
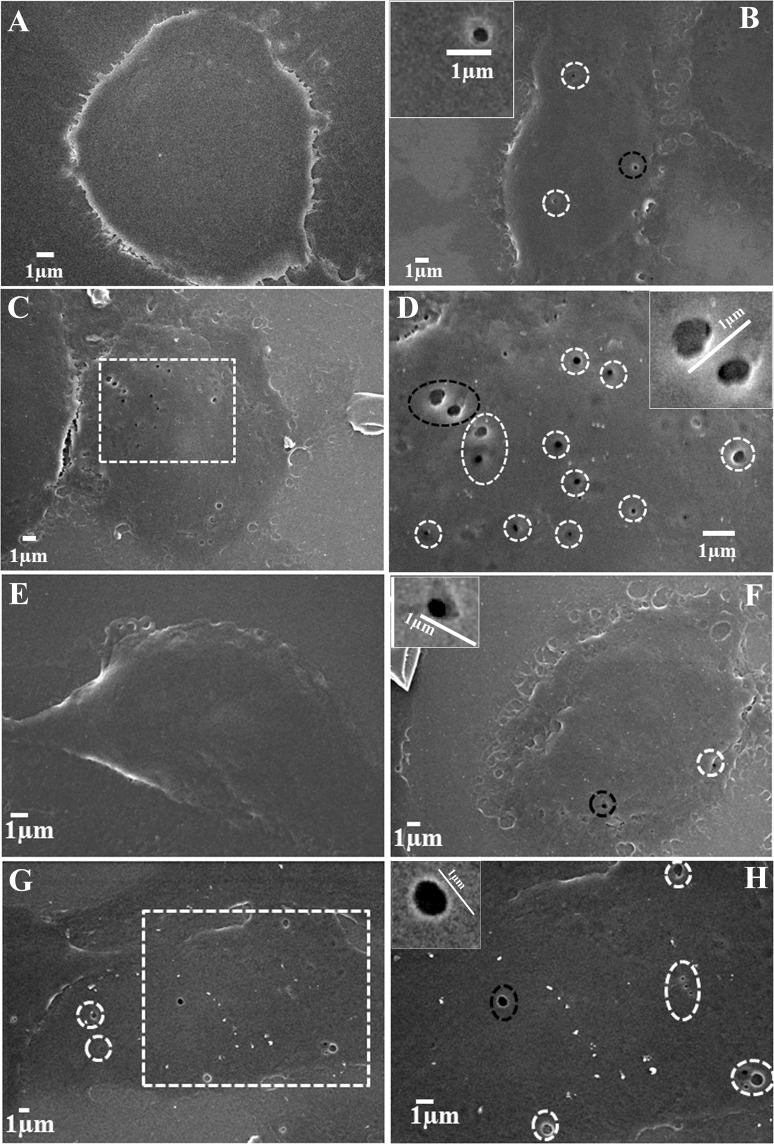
Sonopores developed in the plasma membrane of cells after treatment with ultrasound in the presence and absence of MB (**A**) untreated MDA MB 231 cells (**B**) ultrasound treatment in absence of MB, dotted circles show pores. The black circled pore is shown in the inset (**C**) ultrasound treatment in the presence of MB, a magnified view of the dotted area is shown in the image (**D**). (**D**) Dotted circles surround pores and black circled pore is shown in the inset. B16F10 melanoma cells (**E**) untreated (**F**) treated with only ultrasound, (**G**) treated with ultrasound in presence of MB; the dotted area is magnified in image H, (**H**) dotted circles surround pores and black dotted circle pore is shown in the inset.

**Figure 5:**
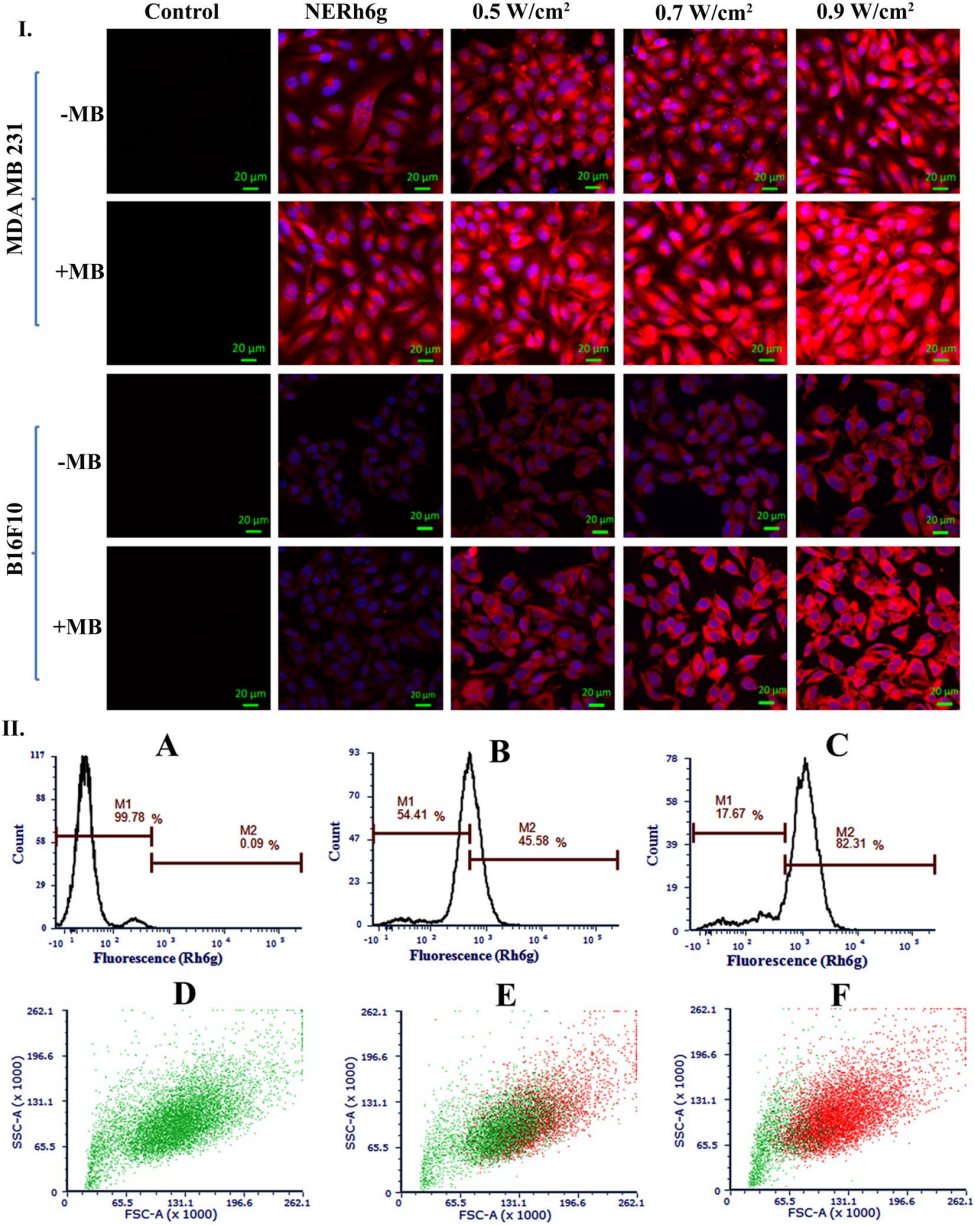
I. CLSM image of NERh6g internalized B16F10 melanoma and MDA MB 231 breast cancer cells. Cells were treated with different intensity of ultrasound in the presence and absence of MB: control, only NERh6g, NERh6g + 0.5 W/cm^2^, 0.7 W/cm^2^, and 0.9 W/cm^2^. II. Internalized NERh6g, by MDA MB 231 cells, was quantified by flow cytometry. Ultrasound treatment enhanced the amount of NERh6g inside cells. M1 indicates the NERh6g negative cells whereas M2 indicates NERh6g positive cells. In the lower panel, green dots indicate cells that are negative for NERh6g whereas the red dots are representing cells that are positive for the NERh6g. (**A**,**D**) Only cells, (**B**,**E**) cells were treated with NERh6g without ultrasound, (**C**,**F**) cells were treated with NERh6g and exposed to ultrasound at 0.9 W/cm^2^ intensity and 50% duty cycle for 30 sec.

SEM analysis of the cells treated with ultrasound in presence of MB revealed that insonation led to the opening of the cell membrane and also the addition of MB further amplified the process of pore generation in terms of both number/cell as well as the diameter. The number of pores/cell was limited to 2–3 pores (Fig. 4B,F) in case of only ultrasound treatment (in ≈ 85% of cells, a total of 100 cells were analyzed) but in presence of MB same number increased up to 8–12 pores/cell (Fig. 4C,D,G,H) in ≈90% of the cells. As far as the diameter of the sonopores is concerned, the diameter of pores generated through ultrasound solely was restricted to 100–300 nm (Fig. 4B,F) whereas in the presence of MB, the width of the pores increased up to 500–700 nm, as shown in Fig. 5C,D,G,H. The observation was similar in both B16F10 and MDA MB 231 cells. Moreover, another interesting finding was breaching (≈1 µm wide) of the cell membrane in few cells due to severe disintegration of membrane following insonation in presence of MB (see Supplementary Fig. S8). As per the literature, opening of membrane due to insonation is transient in nature, so the limitation of our experiment is that it can give a comparative aspect of the effect of ultrasound exposure over cells in presence and absence of MB as by the time we fixed the cells some of the pores would have resealed themselves.

### Cellular internalization of NERh6g with ultrasound

Cellular internalization study of NERh6g indicates that the exposure of ultrasound in the presence of MB increased the cellular uptake as we observed in CLSM analysis of MDA MB 231 and B16F10 cells (Fig. 5I). Moreover, the gradual increase in the intensity of ultrasound viz. 0.5, 0.7 and 0.9 W/cm^2^ increased the fluorescence intensity inside cells which could have been possible due to enhancement in the internalization of NERh6g (Fig. 5I). The addition of MB further enhanced the process of internalization as it provided a higher intracellular intensity of the rhodamine 6g (Rh6g) inside the cells at above-mentioned ultrasound intensity (Fig. 5I). The fluorescence intensity of the internalized amount of NERh6g was quantified by flow cytometry also and found to be increased 3-fold in case of ultrasound treatment as compared to control where only NERh6g was incubated with cells (Fig. 5IIA–C). Ultrasound application not only enhanced the cellular uptake but also the number of cells involved in internalization. In the case of only NERh6g treatment, 45.58% of cells were positive for the Rh6g but the application of ultrasound made 82.31% of the cells positive for Rh6g (Fig. 5IID–F).

### Cytotoxicity and apoptosis assay

**Figure 6:**
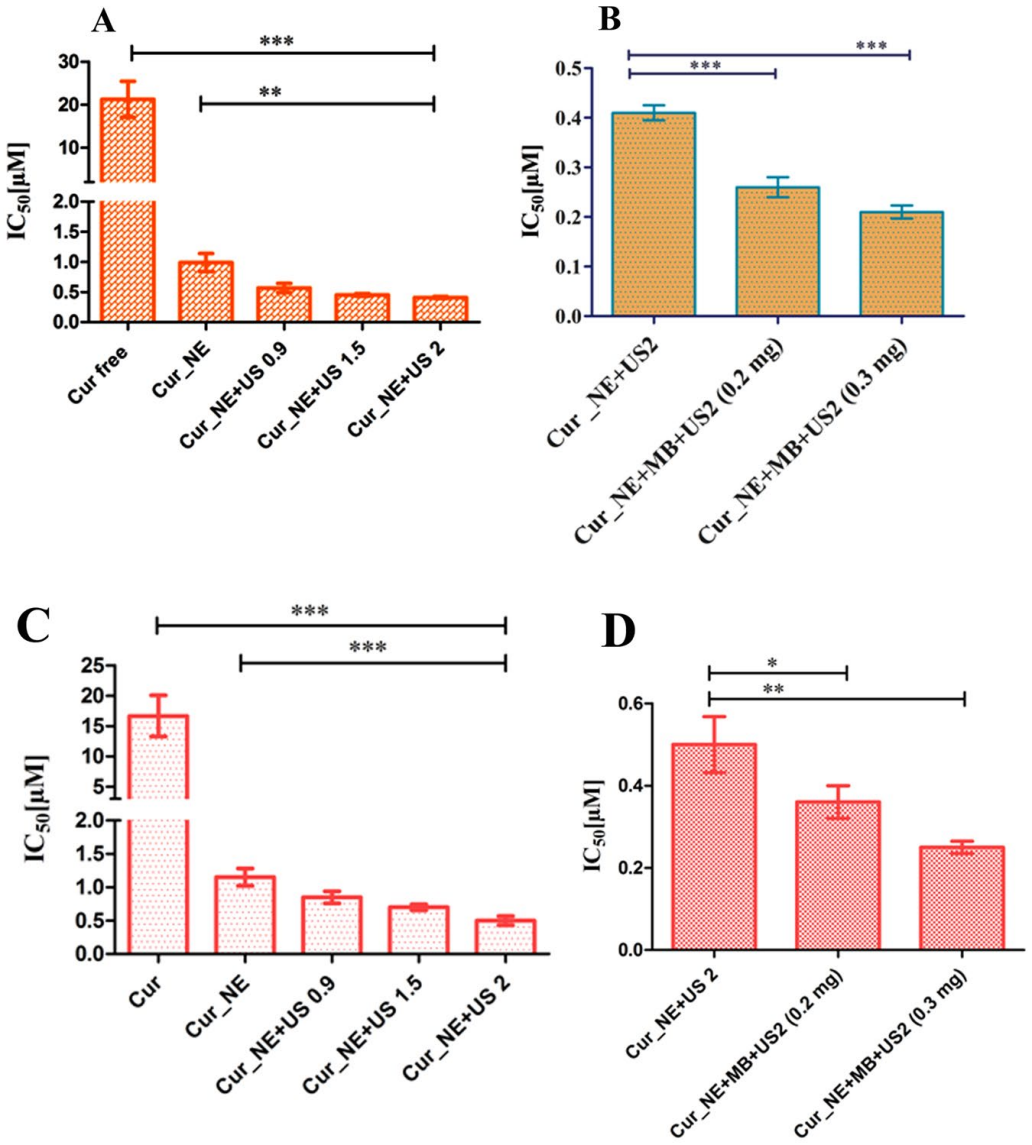
The IC_50_ concentration of the Cur_NE following ultrasound exposure at intensity 0.9, 1.5 and 2 W/cm^2^ in (**A**) MDA MB 231 cells, **P ≤ 0.01, ***P ≤ 0.001 (**C**) B16F10 cells, ***P ≤ 0.001. The IC_50_ concentration of the Cur_NE at ultrasound intensity 2 W/cm^2^ in presence of 0.2 and 0.3 mg of MB in: (**B**) MDA MB 231, ***P ≤ 0.001 (**D**) B16F10 cells, *P ≤ 0.05, **P ≤ 0.01. Data are presented in the form of mean ± SD and P value was determined by Student *t-test*.

Cytotoxicity of the Cur_NE was determined both with and without the assistance of MB in MDA MB 231 and B16F10 cells. As was observed in the CLSM study, NERh6g internalization was enhanced by ultrasound with and without MB and so did the cytotoxicity, as summarized in Table 3. Mere encapsulation of the curcumin inside nanoemulsions reduced the IC_50_ of the drug by 21-fold and further application of ultrasound over MDA MB 231 cells at intensity 0.9, 1.5 and 2 W/cm^2^ reduced the IC_50_ by 36-, 46-, and 51-fold, respectively, as compared to Cur, as shown in Fig. 6A. Cur_NE + US 0.9, Cur_NE + US 1.5, and Cur_NE + US 2 refers to the ultrasound treatment in the absence of MB at intensity 0.9, 1.5 and 2 W/cm^2^, respectively, for 30 sec at 50% duty cycle. Similarly, in B16F10 cells, the IC_50_ value of Cur_NE, Cur_NE + US 0.9, Cur_NE + US 1.5 and Cur_NE + US 2 was found to be 14-, 19-, 23-, and 33-fold, respectively, lower to the Cur (Fig. 6C). In another set of experiment, at the same concentration of Cur_NE, 0.2 mg and 0.3 mg/ml of MB was added to the solution followed by ultrasound treatment at 2 W/cm^2^ for 30 sec with 50% duty cycle. IC_50_ value reduced by 1.6- and 2-fold after treatments with Cur_NE + US2 + MB (0.2) and Cur_NE + US2 + MB (0.3) respectively as compared to Cur_NE + US2 in MDA MB 231 cells, as shown in Fig. 6B. In B16F10 cells, the IC_50_ value of Cur_NE + US 2 reduced by 1.4- and 2-fold due to the addition of MB at concentration 0.2 and 0.3 mg/ml, respectively (Fig. 6D). Apoptosis assay showed that the presence of MB and ultrasound increased the population of early and late apoptotic MDA MB 231 cells (see Supplementary Fig. S9).

**Table 3:** IC50 of the Cur_NE in cancer cells.

Drug /Nanoemulsions	IC50 (µM) in MDA MB 231	IC50 (µM) in B16F10
Cur	21.25 ± 4.2	16.67 ± 3.4
Cur_NE	0.99 ± 0.15	1.15 ± 0.13
Cur_NE + US 0.9	0.57 ± 0.07	0.85 ± 0.09
Cur_NE + US 1.5	0.45 ± 0.02	0.70 ± 0.045
Cur_NE + US 2	0.41 ± 0.01	0.50 ± 0.07
Cur_NE + MB + US2 (0.2 mg)	0.26 ± 0.02	0.36 ± 0.04
Cur_NE + MB + US2 (0.3 mg)	0.21 ± 0.013	0.25 ± 0.015

### Pharmacokinetic study

**Figure 7:**
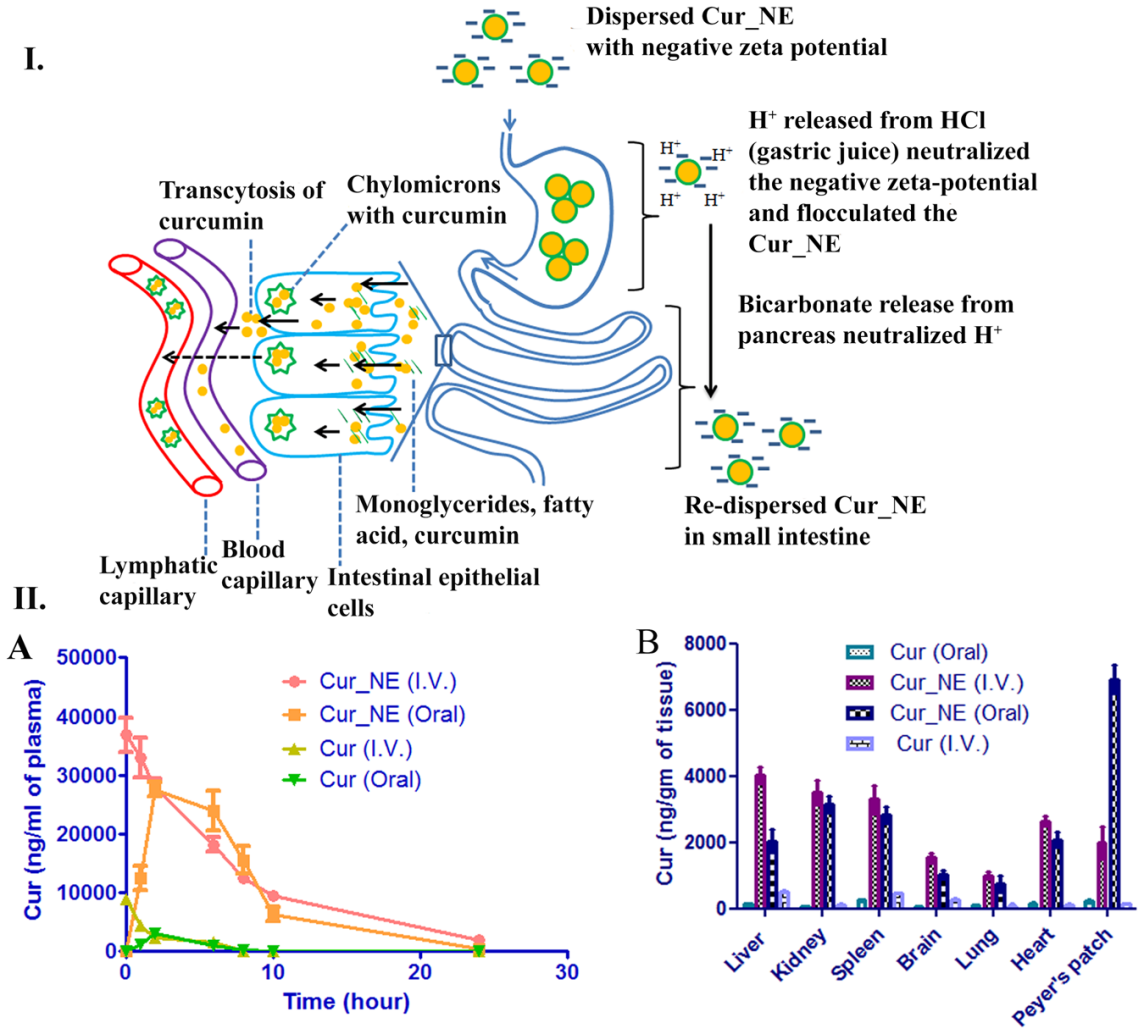
Schematic presentation of the mechanism of absorption of the Cur_NE in the stomach and small intestine. The figure also shows the aggregation of Cur_NE in the stomach and intestinal absorption of the same in either free form or inside chylomicrons. II. (**A**) The concentration of curcumin in plasma following oral and intravenous administration of formulations. (**B**) Biodistribution of curcumin in vital organs after oral and intravenous administration of formulations. Cur (I.V.) and Cur (Oral) stands for intravenous and oral administration of free curcumin.

The pharmacokinetic study showed that the encapsulation of curcumin inside nanoemulsions increased the total drug exposure amount (AUC_0-∞_), elimination half-life (T_1/2_), peak plasma concentration (C_max_) and biodistribution of the drug in various vital organ (Fig. 7IIA,B). In case of oral administration, the T_1/2,_ C_max_ and AUC_0-∞_ for free curcumin was 1.5 ± 0.55 h, 3.1 ± 0.65 µg, and 12.9 µg.h/ml, respectively, but its encapsulation inside nanoemulsions raised them to 5.7 ± 1.2 h, 27.5 ± 4.5 µg, and 239.5 ± 7.5 µg.h/ml, respectively. In the case of intravenous administration, T_1/2,_ C_max_ and AUC_0-∞_ of free curcumin was 2.1 ± 0.3 h, 8.8 ± 0.7 µg, and 21.4 ± 1.2 µg/ml.h, respectively, but the encapsulation inside nanoemulsions increased the pharmacokinetic parameters to 8.5 ± 2.3 h, 37 ± 3.4 µg, and 304.1 ± 8.4 µg.h/ml, respectively (Table 4). The absolute bioavailability of curcumin was calculated [(AUC_0-∞ Cur_NE oral_/AUC_0-∞ Cur_NE I.V._) × 100] for single dose administration of Cur_NE both orally and intravenously with concentration 10 mg/kg body weight of curcumin and was found to be 78.61%.

**Table 4:** Elimination half- life (T_1/2_), peak plasma concentration (C_max_) and total drug exposure (AUC_0-∞_) of curcumin.

Nanoemulsions/drug	T_1/2_ (h)	C_max_ (µg)	AUC_0-∞_ (µg.h/ml)
Cur_NE (Oral)	5.8 ± 1.2	27.5 ± 4.5	239.5 ± 7.5
Cur_NE (I.V)	8.5 ± 2.3	37 ± 3.4	304.1 ± 8.4
Cur (Oral)	1.5 ± 0.6	3.1 ± 0.6	12.9 ± 3.5
Cur (I.V)	2.1 ± 0.3	8.8 ± 0.7	21.4 ± 1.2

### Anti-tumor efficacy

**Figure 8:**
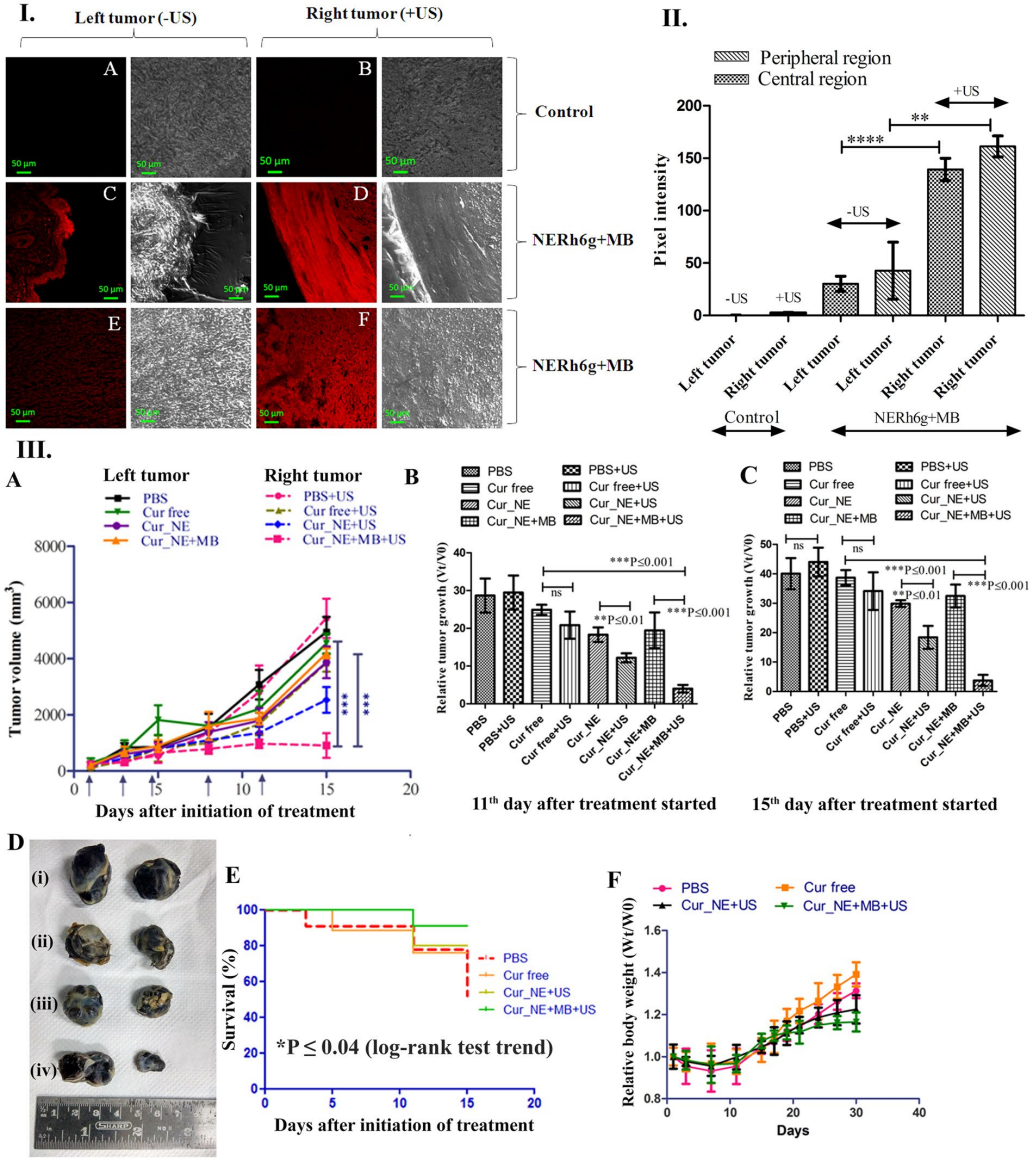
I. Ultrasound exposure over the right tumor spatially enhanced the uptake of the NERh6g and CLSM image of a thin section of the tumors. Red colour fluorescence is due to the internalization of NERh6g by tumor, (**A**) central region of the tumor (**B**) peripheral region of the tumor (**C**) peripheral region of the left tumor which was not exposed to ultrasound (**D**) peripheral region of the right tumor which was exposed to ultrasound for 60 sec at intensity 2 W/cm^2^, duty cycle of 50%, (**E**) central region of the left tumor, (**F**) central region of the right tumor. II. The average pixel intensity of the red fluorescence from each image was calculated and plotted. Ten points were considered from each image for calculating pixel intensity. P value was calculated by Student *t-test*, and values are presented in the form of mean ± SD. ****P ≤ 0.0001 and **P ≤ 0.01. III. Antitumor efficacy of the Cur_NE in the presence and absence of MB injection. The right tumor was exposed to ultrasound whereas the left tumor was kept unexposed. (**A**) A graph is showing change in tumor volume over time after oral administration of formulations. (**B**,**C**) Relative tumor growth was observed over the period. (**D**) Image of tumors from different groups at the end of treatment (30^th^ day), (i) PBS (ii) Cur free (iii) Cur_NE (iv) Cur_NE + MB. (**E**) Kaplan-Meier curve, percent survival of mice in different groups of treatment (Log-Rank test trend *P ≤ 0.04). (**F**) Change in average body weight in each group over the entire period of study. P value was calculated by Student *t- test*, and data are presented as mean ± SD.

The anti-tumor efficacy study illustrated that the application of ultrasound reduced the tumor volume and relative growth rate of the tumor. In most of the groups, the volume of the right tumor (insonated) was significantly (P ≤ 0.01, P ≤ 0.001) smaller than the left tumor which was not exposed to ultrasound, as shown in Fig. 8IIIA,D, except the group treated with PBS and free curcumin (P = 0.2, P = 0.06). When mice were given oral administration of only Cur_NE and exposed with ultrasound, the right tumor showed 1.6-fold higher growth inhibition as compared to the left tumor on the 15^th^ day after treatment started (Fig. 8C). The oral administration of Cur_NE followed by I.V. injection of MB after 2 hrs and ultrasound exposure over right tumor reduced the relative growth rate of the tumor by 11-fold (P ≤ 0.001) compared to the group treated with free curcumin at the end of 15^th^ day after treatment started (Fig. 8C). Ultrasound exposed right tumor had shown a substantial reduction in growth rate as compared to the left tumor of the same mice. It was also proved in another experiment where mice were given an oral dose of NERh6g nanoemulsions and after 2 hrs, MBs were injected and the right tumor was exposed with ultrasound whereas the left tumor was kept unexposed. The CLSM analysis of the thin section of tumors showed that the intensity of Rh6g in the right tumor was substantially higher than the left tumor (Fig. 8I,II). Although, the distribution of Rh6g was decreasing from periphery to center still the decrease in intensity was not substantial (Fig. 8I,II). In the peripheral region, the Rh6g intensity of the right tumor was 4-fold higher than the corresponding region of the left tumor (Fig. 8I,II). In the central region also, the intensity of Rh6g was 4.5-fold higher in the right tumor as compared to the corresponding region in the left tumor (Fig. 8I,II). Moreover, in Kaplan-Meier survival study it was found that during treatment the maximum percentage (90.9%) of animal survived in a group treated with Cur_NE + MB + US whereas the least survival percentage (51.9%) was observed in a group treated with PBS (Fig. 8III,E). The group treated with free curcumin, 75.6% (Fig. 8IIIE) of animals survived at the end of treatment schedule whereas the group Cur_NE + US showed a survival percentage of 80%. Kaplan-Meier study showed a significant (log-rank test, P ≤ 0.04) trend of increase in survival percentage. As far as the decrease in body weight of mice is concerned, in all the groups, relative body weight decreased initially post cells injection but soon it increased gradually (Fig. 8IIIF). Once treatment started, the decrease in body weight followed the trend of PBS > Cur free > Cur_NE > Cur_NE + MB. It suggests that the decrease in body weight occurred due to the decrease in tumor volume as a decrease in both, body weight as well as tumor volume, followed the same pattern.

## Discussion

Cur_NE from different ratios of lecithin and curcumin were developed and characterized. It was found that at all the ratios provided spherical shaped stable Cur_NE with diameter 60–120 nm and optimum negative zeta potential. Moreover, the ^1^H NMR spectrum revealed that the process of encapsulation was not altering the chemical structure of the curcumin and was stable inside the Cur_NE. An increase in the concentration of curcumin at a fixed concentration of lecithin eventually saturated the loading capacity. The ratio 2:1 showed higher internalization as compared to 1.5:1 and 1:1, as the hydrophilicity of latter nanoemulsions (1.5 & 1:1) would have decreased compared to the former one (2:1) due to the gradual increase in filling of curcumin. Hence, ratio 2:1 was preferred over all the other ratios for further studies. The developed nanoemulsions was biocompatible with normal fibroblast cell line (see Supplementary Fig. S12). Solubility assay revealed that the encapsulation of the curcumin inside lecithin nanoemulsions turned the whole nanostructure hydrophilic and that is why all the ratios got easily resuspended in PBS but the same amount of free drug was highly hydrophobic and could not be dispersed in PBS.

The developed MBs were proved to be echogenic i.e. capable of generating ultrasound contrast (see Supplementary Fig. S5). Application of ultrasound over cells in presence of MB lead to the cavitation of latter which in turn exerted shear stress over the cells situated nearby and generated small pores in the plasma membrane as observed in Fig. 4 in both MDA MB 231 and B16F10 cells. Cells treated with ultrasound developed small pores in the plasma membrane but pores generated after ultrasound treatment in presence of MB were higher in count/cell and also larger in diameter compared to ultrasound treatment in absence of MB. Moreover, it was found that if MBs cavitated and burst near the cell, it created the pore with a wide diameter (≈1 µm) due to an extreme shear force and jet propulsion around cells (see Supplementary Fig. S8). These pores facilitated the intracellular delivery of therapeutics.

That is why the addition of MB further enhanced the Rh6g intensity inside the cells as compared to the ultrasound alone and hence the ultrasound application will be very crucial in increasing cytotoxicity of a drug. The insonation also increased the population of cells involved in internalization and hence the application of ultrasound would ensure the maximum participation of the constituent cells of tumor in the uptake of drug and therefore maximum regression of the tumor. Through the same mechanism, the internalization of Cur_NE would have enhanced in the presence of ultrasound and MB, which resulted in a substantial reduction in IC_50_ of curcumin. These sonopores greater in number and diameter would have ensured the higher uptake of curcumin to reduce the IC_50_ and even after the expulsion of the drug through pgp receptor, enough amount of drug would have been inside the cells to stop the cellular proliferation (see Supplementary Fig. S10). Moreover, the insonation of cells; in the presence as well as absence of MB, increased the percentage of apoptotic cells. It is already reported that the application of low intensity pulsed ultrasound (>0.5 W/cm^2^) substantially increased the intracellular Ca^2+^ concentrations and expression of caspase 3, Bcl-2 and Bax which in turn increased the apoptosis of hepatocellular carcinoma cells. Ca^2+^ dependent expression of apoptotic inducer and curcumin would have synergistically increased the population of late apoptotic cells^27^. Moreover, the increased uptake of curcumin inside cells due to presence MB and ultrasound would have enhanced the signal cascade responsible for the apoptosis of cells. Park *et al*. 2013 reported that curcumin enhanced the TNF related ligand-induced apoptosis in breast cancer cells MCF-7, T47D and SK-BR-3^28^.

Release kinetics study in SGF and SIF revealed that the formulation is resistant towards the gastric juice of stomach but sensitive to the intestinal fluid. Hence, it will not release the curcumin in the extreme acidic condition of gastric juice but will be easily digested in the small intestine and enhance the absorption of the curcumin. It was observed that the Cur_NE got agglomerated in SGF but the addition of NaOH (increased the solution’s pH 6.8–7.4) re-dispersed the Cur_NE completely and intact Cur_NEs were present in solution (Fig. 3F,G)^29^. Naturally, the bicarbonate released from the pancreas will play a role in this neutralization of HCl. Hence, when Cur_NE will cross the stomach, the pH of the small intestine would re-disperse the Cur_NE again in colloidal form (Fig. 3F). Moreover, the re-dispersion would expose the individual particles to pancreatin and facilitate the enzymatic digestion of Cur_NE to release free fatty acid, monoglycerides, and curcumin. Thereafter, this free curcumin would be absorbed either alone or in combination with monoglycerides and fatty acid in the form of chylomicrons. Curcumin released from Cur_NE would have entered the circulation presumably through two pathways: (i) transcytosis of the curcumin through intestinal epithelial cells and, (ii) chylomicrons encapsulated with curcumin diffuses to the lymphatic system and then to blood circulation (Fig. 7I)^30,31^. Moreover, the higher concentration of curcumin in Peyer’s Patch suggests that the Cur_NE got absorbed through the route of the first-pass metabolism and entered the circulation through the lymphatic capillary in the form of chylomicrons. Consequently, T_1/2_, C_max_, and AUC_0-∞_ as well as biodistribution of the curcumin in vital organs increased several-fold.

Effect of ultrasound intensities on fibroblast normal cells showed that intensity of 2 W/cm^2^ did not induce any cell death and more than 90% cell viability was maintained. Also, the histopathology examination of the insonated skin revealed that the ultrasound stimulus, at 2 W/cm^2^ and 50% duty cycle for 60 sec, was safe for topical application as not induced inflammation or ulceration in the skin (see Supplementary Fig. S11). Furthermore, one obvious observation was that in every group right tumor was smaller in size as compared to the left tumor which is attributed to the exposure of ultrasound that increased the uptake of curcumin from extracellular region to inside the cell due to sonoporation. Although the reduction in the tumor volume was not found to be substantial in left tumors in all the treatment. This might have happened due to less uptake of curcumin by the tumor in absence of ultrasound; on the contrary, the exposure of ultrasound over the right tumor enhanced the intracellular delivery of the drug to the intratumoral space. This was evident from a result where we demonstrated that the exposure of ultrasound stimulus could increase the delivery of dye in the region of interest. That is why a substantial reduction in volume observed in the tumor exposed to ultrasound.

Moreover, the repeated use of ultrasound in the presence of MB created sheer stress that led to the death of the cells and that is why even in the absence of any drug, at the end of 15 days, reduction in right tumor occurred in a group treated with PBS, although reduction in tumor was not statistically significant (P = 0.2). Hence, simple ultrasound exposure may not be able to regress the tumor effectively. As per the literature, ultrasound exposure also generates ROS, and its repeated exposure might have produced enough amounts of ROS which led to the death of cancer cells^32^. Hence, the oral delivery of the formulation followed by MB injection and ultrasound treatment provides a unique modality of oral chemotherapy for the treatment of cancer.

## Methods

### Preparation of Cur_NE

The Cur_NEs were prepared by the solvent displacement method. Lecithin and curcumin were dissolved in 27:1, 9:1, 6:1, 4:1, 3:1, 2:1, 1.5:1 and 1:1 wt/wt ratio in 10 ml of organic solvent mixture comprised 1:1 ratio (v/v) of acetone and chloroform; and added slowly into 20 ml of water under the stirring condition of 1200–1300 rpm. This solution was further homogenized at very high pressure of 10000–15000 psi in a homogenizer (AVESTIN EMULSIFLEX C3 homogenizer). The solvent evaporation was followed by isolation of Cur_NE through centrifugation (20000 xg for 30 min). The supernatant thus obtained was used to estimate the amount of curcumin encapsulated inside Cur_NE. To synthesize rhodamine 6g (Rh6g) encapsulated nanoemulsions (NERh6g), 1 mg of Rh6g dye was dissolved in 5 ml chloroform along with 80 mg of lecithin and then 5 ml acetone was added. The rest of the process was similar to the preparation of Cur_NE.

### Preparation of MB

MBs were prepared by thin-film hydration method using DSPC: DOPS-Na: TPGS in 20:1:1.5 molar ratio. A detailed protocol was reported by our group^25^.

### Encapsulation efficiency

Encapsulation efficiency of nanoemulsions was estimating by evaluating drug in supernatant after centrifugation using following formula:

$$EE\,({\rm{ \% }})=[(Total\,drug\,taken\,(mg))\,-\,(Drug\,in\,supernatant\,(mg))/Total\,drug\,taken\,(mg)]\,\ast \,100$$

All drug estimations were done using HPLC (see Supplementary Information S2).

### DLS & Zeta potential

The hydrodynamic diameter was determined using dynamic light scattering (BROOKHAVEN) at 90° angle, 27 °C temperature, and 632 nm wavelength. Zeta potential was determined using Zeta Potentiometer (BROOKHAVEN) at 27° C. Likewise, MBs were diluted appropriately and then analyzed for hydrodynamic diameter and undiluted samples were used for the zeta potential measurement (see supplementary information S.1).

### Cryo FEG SEM

Cur_NE were also characterized through cryo field emission gun scanning electron microscope (FEG-SEM) for diameter and topology. The samples were diluted appropriately and then frozen in liquid nitrogen for 10–15 min and sublimed at −90 °C for 10 min. The sublimed samples were coated with platinum at 10 mA for 45 sec before the imaging.

### NMR

Two milligrams of Cur_NE, curcumin, and lecithin were dissolved in CDCl_3_ and then they were analyzed through 500 MHz ^1^H NMR (5 mm probe, 500 MHz, BRUKER, SWITZERLAND) spectroscopy at 25 °C.

### Solubility assay

The Cur_NE and free curcumin (Cur) were weighed at the same concentration and resuspended in 2 ml of PBS. Thereafter, the colloidal dispersibility of the solution was observed.

### Release kinetics in simulated gastric fluid (SGF) and simulated intestinal fluid (SIF)

The drug retention ability of the Cur_NE in the stomach was evaluated by monitoring the release profile over 48 hrs in SGF. SGF (pH 1.5–2) was prepared as reported previously after making desired changes in the protocol^33^. In short, 0.2% NaCl and 0.32% pepsin were dissolved in 7 ml HCl and finally, volume was adjusted to 1000 ml. The release medium comprised 0.5% (v/v) tween 80 in SGF. Cur_NE, with concentration 1 mg/ml curcumin, was packed inside dialysis bag (HIMEDIA LAB 387 Dialysis Membrane-50, molecular wt. cut-off 12,000 to 14,000) and kept in sink condition with the required volume of release medium in USP dissolution apparatus type-II (ELECTROLAB, TDT-08L). The amount of released curcumin was estimated at different time points 0, 0.25, 0.5, 1, 2, 4, 6, 8, 12, 24, 36 and 48 hrs using HPLC at 420 nm after making appropriate dilutions.

Release kinetics was also studied in SIF with pH 6.8–7.4 and was performed similar to the release kinetics in SGF. The composition of the SIF was preferred as reported by USP and changes were made in the protocol as per requirement. Briefly, 0.05 M monobasic potassium phosphate, 0.15 M NaOH, 3 mM sodium taurocholate and 1% w/v of pancreatin at pH 6.8^33^. The release medium comprised 0.5% (v/v) of tween 80 in SIF.

### Cell culture

All cell culture experiments were performed inside laminar air flow in aseptic conditions (THERMOSCIENTIFIC) and incubated at 37 °C in the humidified environment with 5% CO_2_ (THERMOSCIENTIFIC CO_2_ incubator). MDA MB 231 and B16F10 cells were seeded in 24 and 96 well plates as 1 × 10^5^ and 3 × 10^4^ cells/well respectively in DMEM culture medium supplemented with 10% FBS and 1% antibiotic antimycotic solution. Cell viability was determined by MTT assay^25–34^.

### MB induced sonoporation in MDA MB 231 and B16F10 cells

MDA MB 231 and B16F10 cells were grown overnight in 24 well plates and the study was performed as reported by our group^25^.

### Internalization of Cur_NE

Eighty microgram of the encapsulated drug (in the form of Cur_NE) from each ratio of lecithin: curcumin, as well as free curcumin, was incubated with overnight grown MDA MB 231 cells in 24 wells plate for 1.5 hrs and after that wells washed with PBS. One milliliter of ACN was added to lyse the cells. Twenty microliters of lysate was injected to HPLC column (RP-18, 5 µm, LiChrosorb MERCK) for estimating the amount of curcumin present in cell lysate at 420 nm with continuous flow of mobile phase acetonitrile: methanol: 20 mM acetate buffer at pH 3 (40:30:30, v/v).

### Internalization of NERh6g

CLSM imaging was performed to study the cellular uptake as reported^25^. Briefly, 14 µg NERh6g was added and the cells were treated with ultrasound at a 50% duty cycle for 30 sec with varying intensity viz. 0.5, 0.7, 0.9 W/cm^2^. In another set of experiment, cells were supplemented with 14 µg of NERh6g and 50 µg of MB before exposing with ultrasound at 50% duty cycle for 30 sec at an intensity of 0.9 W/cm^2^. The cells were incubated for 1.5 hrs. After incubation, to stain the nucleus, 0.02 nM of Hoechst 33342 (ANASPEC, Inc.) dye was added and left for 10 min. Likewise, at the same concentration of NERh6g, cellular uptake study was performed by flow cytometry^25^.

### Cytotoxicity in MDA MB 231 breast cancer and B16F10 melanoma cancer cells

MDA MB 231 and B16F10 cells were grown overnight in 96 well plates and fresh medium containing serial dilutions of Cur_NE with curcumin concentration 100, 25, 6.3, 1.6, 0.4, 0.1, 0.02 µM was added. After the addition of Cur_NE, cells were treated with ultrasound at a 50% duty cycle at intensities 0.9, 1.5 and 2 W/cm^2^ for 30 sec. In another set of experiments, the effect of MB on cytotoxicity was observed and in addition to Cur_NE, 0.2 and 0.3 mg/ml of MB was also added and serially diluted. At the end of 72 hrs, MTT assay was performed to determine the cell viability.

### Apoptosis assay

The flow cytometry assay was performed to determine the apoptotic induction ability of the Cur_NE in presence of MB and ultrasound^25^. Cells were treated with Cur, Cur_NE, Cur_NE + US, and Cur_NE + MB + US; the US stands for the ultrasound treatment at intensity 2 W/cm^2^, duty cycle 50% for 30 sec. The concentration of Cur, Cur_NE, Cur_NE + US used for the treatment was equal to the IC_50_ concentration of Cur_NE + MB + US (0.3) and following ultrasound exposure entire tests and control were incubated further for 72 hrs.

### Animal handling

All animal experiments were performed as per guidelines and regulations laid down by Committee for the Purpose of Control and Supervision of Experiments on Animals (CPCSEA) in India. The studies (37/1516 and 08/1718) were approved by the chairman of Institutional Animal Ethics Committee and nominee of the CPCSEA at National Toxicology Centre Pune.

### *In vivo* pharmacokinetic study

The pharmacokinetic study was performed in 6–8 weeks old albino Wistar rats (n = 6) with body weight 200–250 g. Ten mg/kg body weight single dose (oral or I.V.) was administered and after administration, 0.5 ml blood sample was collected through retro-orbital puncture at intervals 0, 1, 2, 6, 8, 10, 24 hrs. Plasma was processed and curcumin was extracted as reported previously^25^. To study biodistribution, the entire organ was weighed and then 0.5 g of organs viz. liver, kidney, spleen, lung, brain, Peyer’s patch and heart were homogenized in 2 ml acetate buffer (pH 5) and the rest of the process of curcumin extraction was similar to the previously reported article^25^.

### *In vivo* anti-tumor efficacy

To study the *in vivo* anti-tumor efficacy, tumors were generated on both the flanks of the mice (C57BL/6, n = 6, 6–8 weeks old) as reported by us^25^. Thereafter, Forty mg/kg body weight of free Cur, Cur_NE was administered orally to the mice of all groups and then after 2 hrs of dosing, 250 µg of MB was injected through the tail vein. Just after injection of MB, ultrasound treatment was given at parameters 2 W/cm^2^, 50% duty cycle for 60 sec to the right tumor whereas the left tumor was kept unexposed. A total of 5 treatments were given, first three on alternate days and last two after a gap of two days. To determine the tumor uptake, 0.5 mg (20 mg/kg body weight) of NERh6g was administered orally to the tumor bearing mice. Then after 2 hrs, 250 µg of MB was injected through the tail vein and just after that right tumor was exposed with ultrasound. Tumors were excised and then analyzed by CLSM^25^.

### Statistical analysis

All the experiments were conducted in triplicates unless stated otherwise. The graphs are showing data in the form of mean ± SD. The statistical significance of the results was calculated by the Student *t-test* (two-tailed model).

## Supplementary information

Supplementary information.
