# Supplementary material for: Curcumin Encapsulated Lecithin Nanoemulsions: An Oral Platform for Ultrasound Mediated Spatiotemporal Delivery of Curcumin to the Tumor
Source: Sci Rep. 2020 May 22;10:8587. doi: 10.1038/s41598-020-65468-1 (PMC7244714; doi:10.1038/s41598-020-65468-1)
Supplement: Supplementary file 1 — Supplementary information. [file 41598_2020_65468_MOESM1_ESM.docx]

**Supplementary Data**

**Curcumin Encapsulated Lecithin Nanoemulsions: An Oral Platform for Ultrasound Mediated Spatiotemporal Delivery of Curcumin to the Tumor**

Chandrashekhar Prasad ^a^, Eshant Bhatia ^a^ and Rinti Banerjee ^a,^ *

^a^ *Department of Biosciences and Bioengineering, Indian Institute of Technology Bombay, Mumbai, India*

**Corresponding author: Department of Biosciences and Bioengineering, Indian Institute of Technology, Bombay, Mumbai, India. Email ID: rinti@iitb.ac.in*

**S.1: Dynamic Light Scattering and Zeta potential measurement**

Both Tpt_NC and MB were characterized for its hydrodynamic diameter and zeta potential by DLS (BROOKHAVEN) and Zeta Potentiometer (BROOKHAVEN) respectively. Intensity correlation functions were measured at 90^0^ angle, 25^0^ C temperature and 632 nm wavelength. Before analyzing the sample through DLS, samples (both MB and Cur_NE) were diluted 10 times in PBS (pH 7.4). In zeta potential measurement, undiluted samples were analyzed (15 mg/ml of Cur_NE, with respect to lecithin and 2 mg/ml of MB, with respect to total amount of DSPC, DOPS-Na and TPGS).

**S.2: Determination of encapsulation efficiency**

The encapsulation efficiency (EE) was determined using HPLC (JASCO International) through indirect method using formula,

$$EE\left( \% \right)=\frac{Amount of drug taken \left( mg \right)-Amount of drug in supernatant (mg)}{Amount of drug taken (mg)}\times100$$

To draw a standard curve; 1, 2, 3, 4, 5, 6 and 7 µg/ml of curcumin solutions (stock solution: 5 mg/ml in methanol) were prepared in the mobile phase. The mobile phase was prepared my mixing of acetonitrile, methanol and 20 mM acetate buffer at pH 3 in appropriate ratio of 40:30:30 (v/v) respectively. Then 20 µl of both, the supernatant and the solution with known concentration of curcumin were injected to the HPLC column (RP-18, 5 µm, LiChrosorb MERK) with a continuous flow of mobile phase at 1 ml/min and photo-diode array detector was set at 420 nm. After plotting the standard curve, the encapsulation efficiency was calculated through extrapolation of standard curve.

***
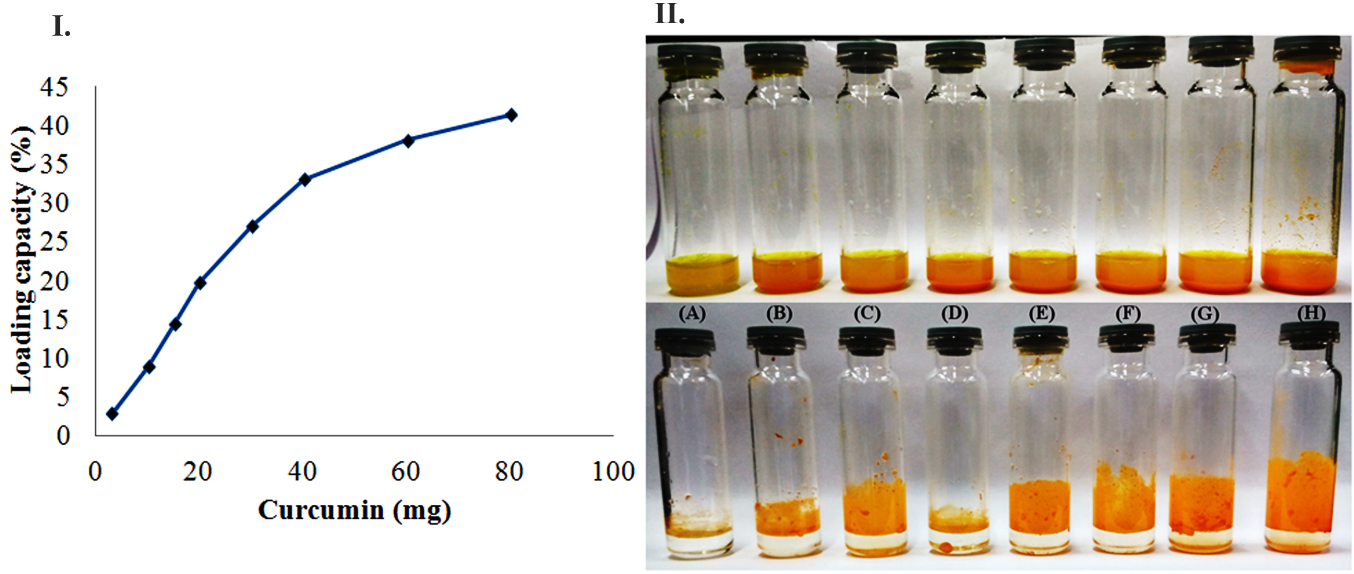
***

**Figure S.3:** (I) Saturation in loading of curcumin with increase in concentration of curcumin at fixed concentration of lecithin. (II) Solubility test of free drug (upper panel) and encapsulated drug (lower panel), (A) 27:1 (B) 9:1 (C) 6:1 (D) 4:1 (E) 3:1 (F) 2:1 (G) 1.5:1 (H) 1:1.

***
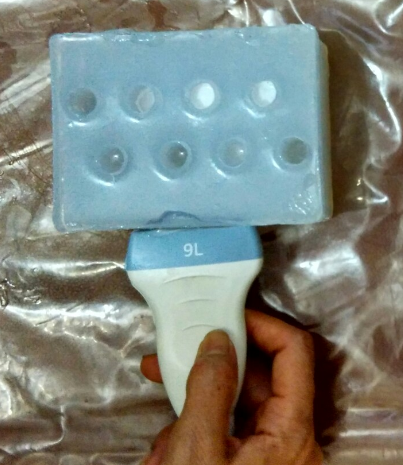
***

**Figure S.4:** Measurement of echogenicity using agarose phantom. It shows an arrangement of the ultrasound probe and its orientation across the well of 1% agarose phantom. 9L ultrasound probe (Agilent Technology) was used to record the image. MB, Sonovue and the degassed PBS was added to the wells at a time.


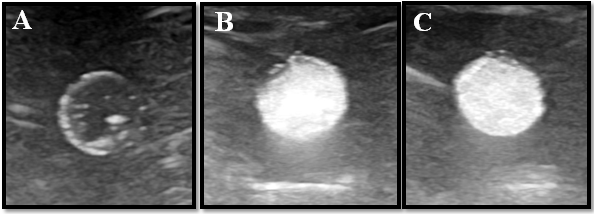


**Figure S.5:** Ultrasound contrast property of the MB and its comparison with the marketed contrast agent sonovue was studied by agarose phantom model. Testing material was added to phantom’s well and B mode ultrasound image was obtained for (A) degassed PBS (B) Sonovue^TM^ (C) MB.


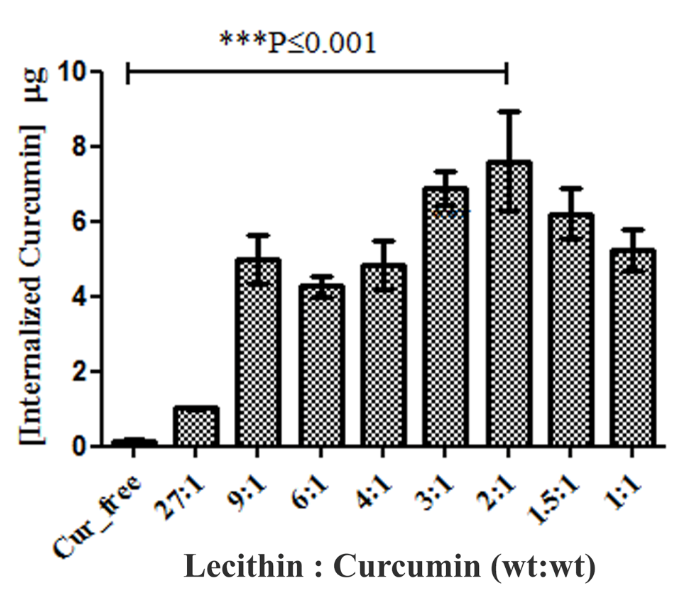


Figure S.6: Comparison of the internalized amount of curcumin when 80 mg free curcumin (Cur) and Cur_NE comprised different ratios of lecithin and curcumin were incubated for 1.5 hr with MDA MB 231 cell.


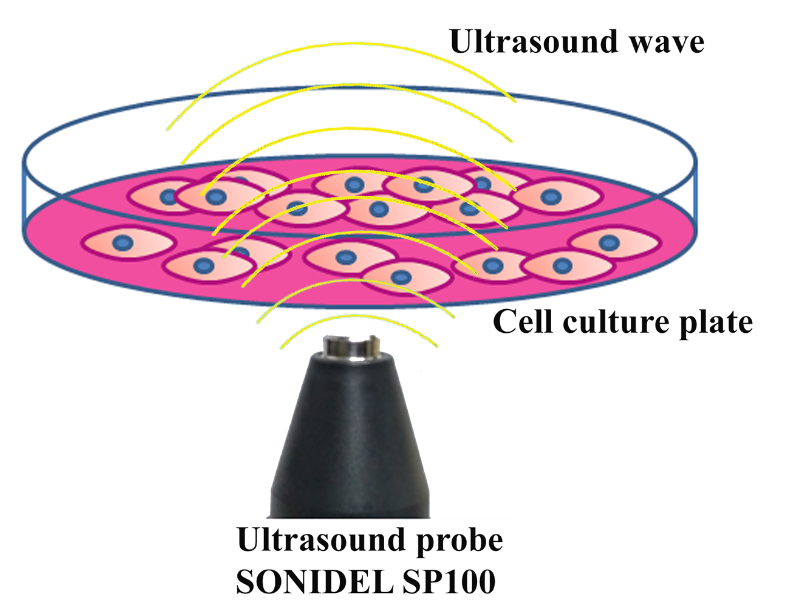


**Figure S.7:** The experimental set up for the sonoporation, internalization and cytotoxicity study**.**


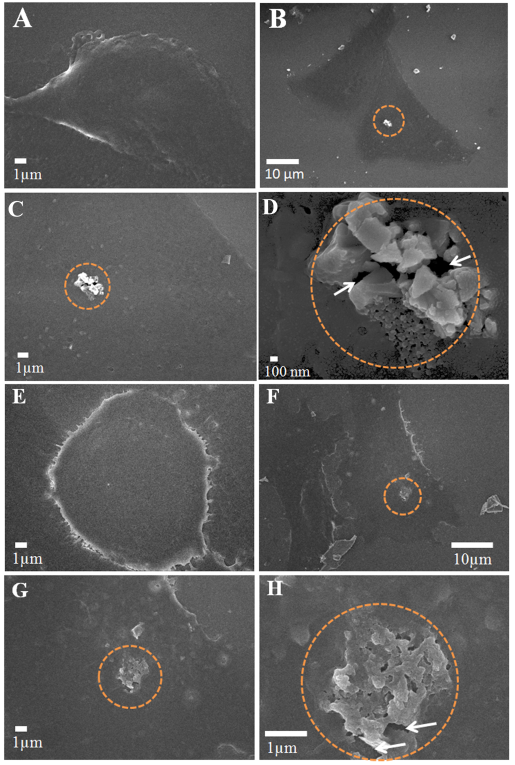


**Figure S.8:** SEM image of cells treated with ultrasound in presence of MB showing generation of pores/breach in the plasma membrane due to bursting/inertial cavitation of MB, (A) B16F10 cells without any treatment, (B) B16F10 cells treated with ultrasound in the presence of MB led to breaching of membrane, (C&D) shows circled area of image B at high magnification. (E) MDA MB 231 cells without any treatment, (F) MDA MB 231 cells treated with ultrasound in presence of MB led to breaching of the membrane, (G&H) shows the circled area of image F at high magnification.


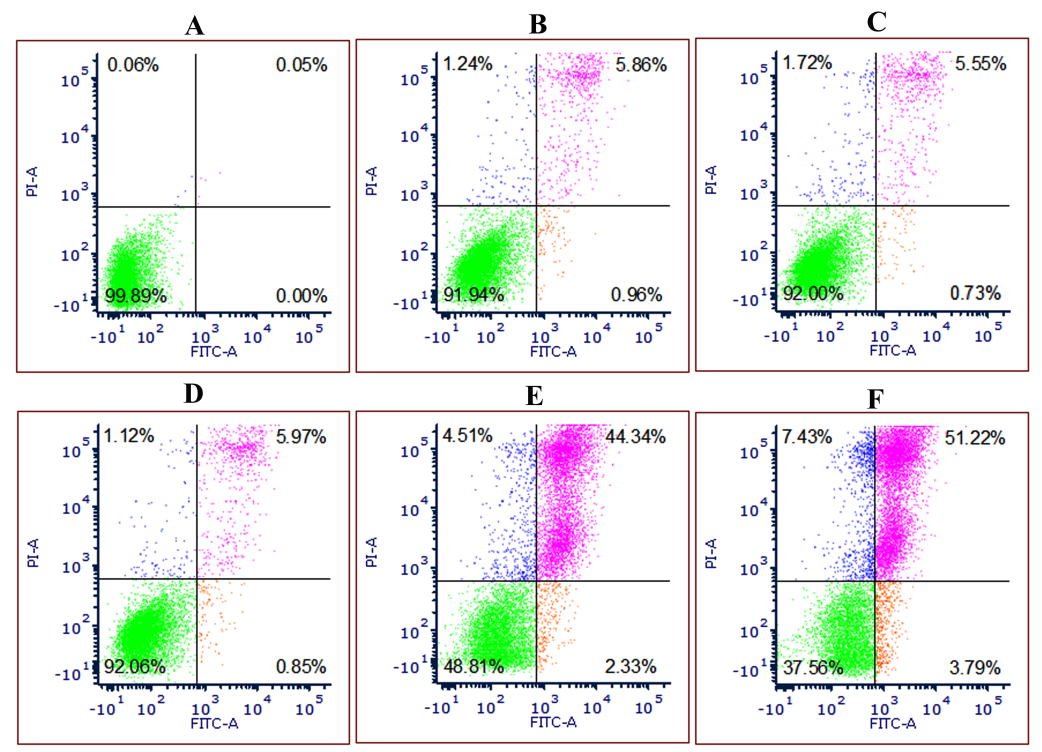


Figure S.9: The amount of live, necrotic, early and late apoptotic MDA MB 231cells are shown in different quadrants in each treatment group, (A) only cells (B) non-treated cells+Annexin V-FITC+PI, (C) free Cur treatment+ Annexin V-FITC+PI, (D) Cur_NE treatment+ Annexin V-FITC+PI, (E) Cur_NE + ultrasound treatment at intensity 2 W/cm^2^ at 50% duty cycle for 30 sec (F) Cur_NE + ultrasound treatment at intensity 2 W/cm^2^ at 50% duty cycle for 30 sec in presence of 0.3 mg of MB.


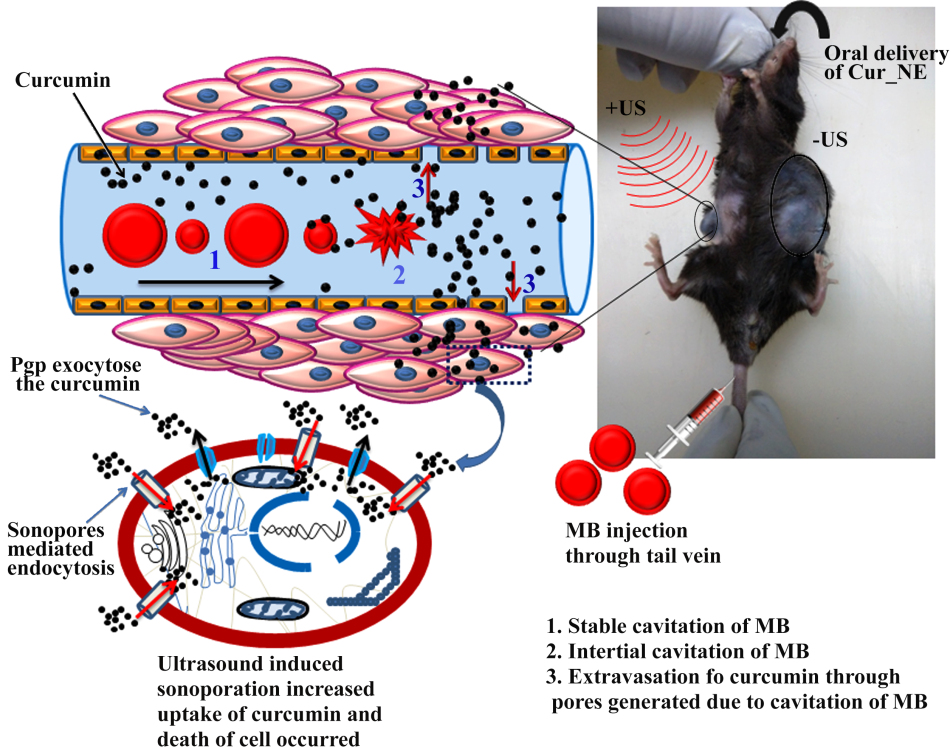


Figure S.10: Schematic representation of the spatial delivery of curcumin. wherein the right tumor exposed to ultrasound regressed significantly as compared to the left tumor that was not exposed to ultrasound. Sonopores mediated internalization outweighed the pgp receptor- mediated exocytosis of curcumin and hence tumor regression occurred.


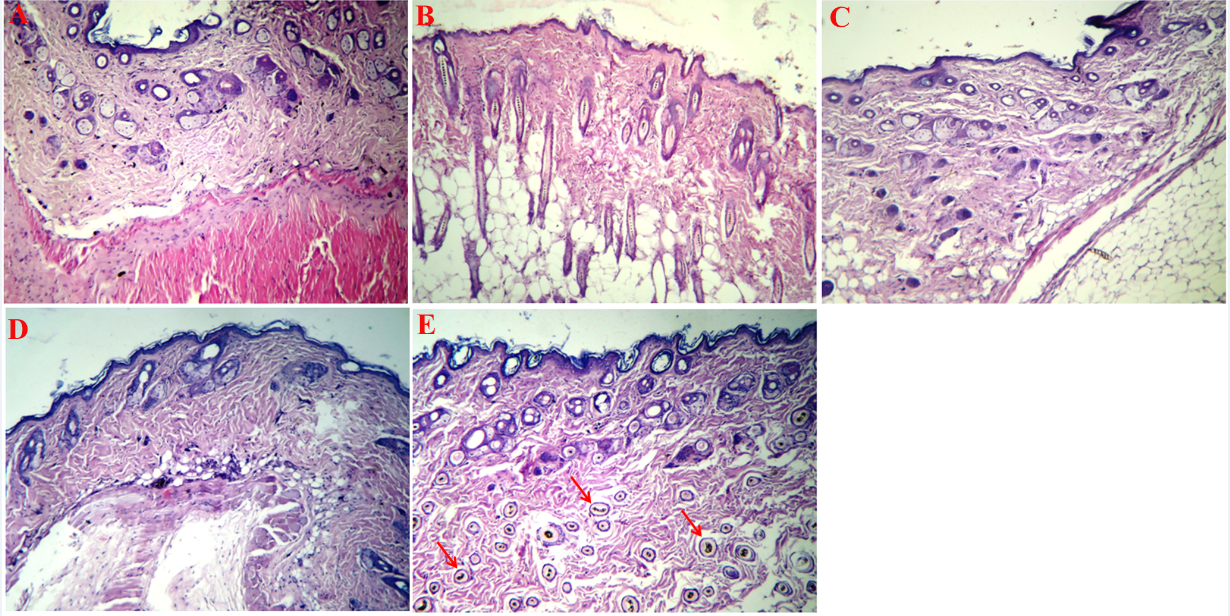


**Figure S.11:** Histopathology results of the skin exposed to ultrasound for 60 sec at 50% duty cycle and varying intensities: (A) control, (B) 1, (C) 2, (D) 3, (E) 4 W/cm^2^. The red arrows in image E indicate infiltrated white blood corpuscles, a sign of mild inflammation.


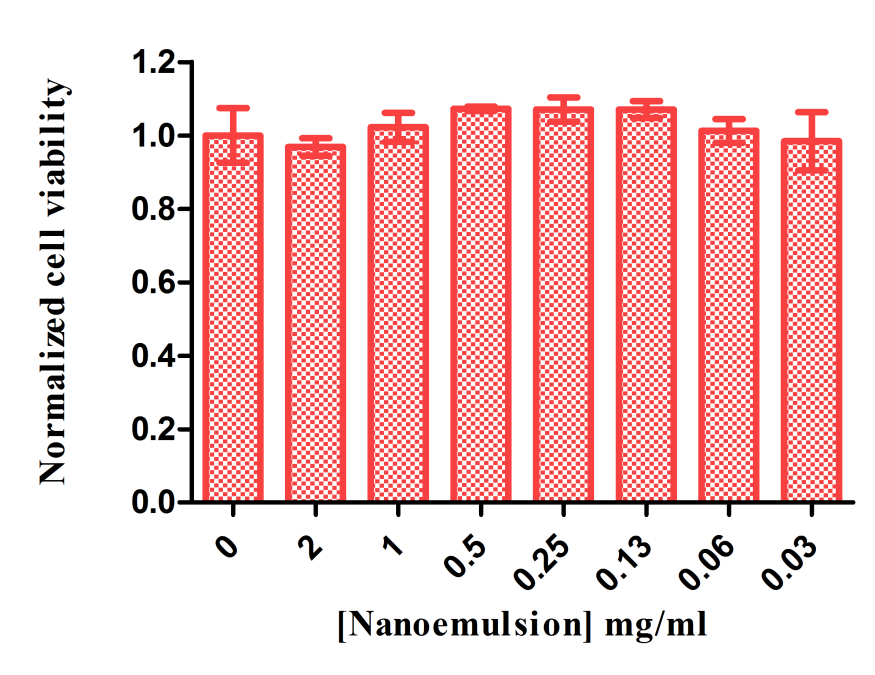


**Figure S.12:** Biocompatibility of nanoemulsions on L929 fibroblast cell line. The data **s**how that the nanoemulsions is compatible with normal fibroblast cells at very high concentrations of 2 mg/ml, indicating them as safe and effective carriers for drug delivery.


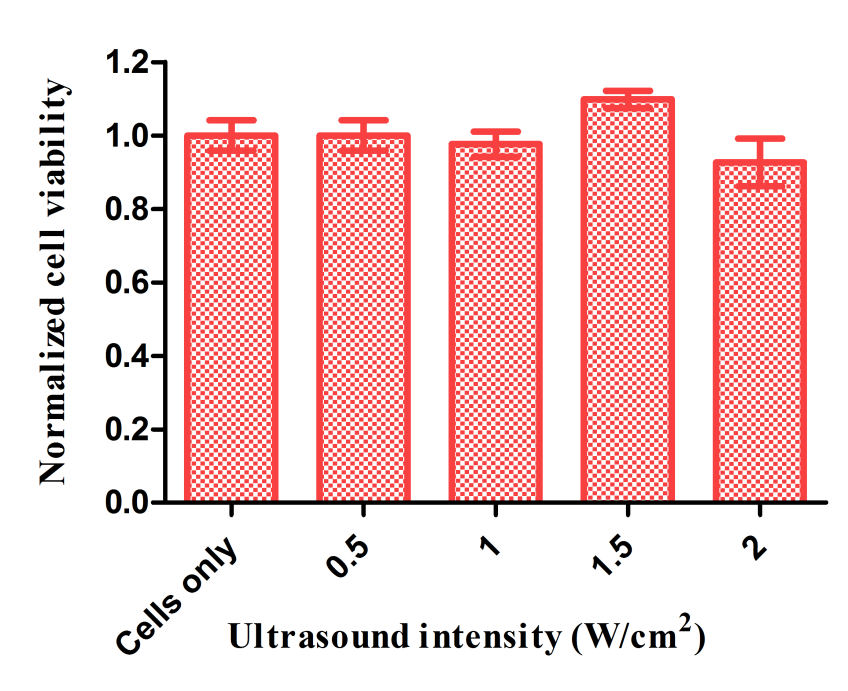


**Figure S.13:** Effect of ultrasound intensities on L929 fibroblast cell line. The data show that ultrasound up to 2 W/cm^2^ is compatible with normal fibroblast cells and did not affect cell viability, thus is safe to use for cancer therapy.
